# Regulation of Metabolic Rhythms by Glial Clocks

**DOI:** 10.1177/07487304251386682

**Published:** 2025-12-04

**Authors:** Catarina Carvalhas-Almeida, Sara B. Noya, Tianqi Wu, Ana Rita Álvaro, Cláudia Cavadas, Julie A. Williams, Amita Sehgal

**Affiliations:** * Center for Neuroscience and Cell Biology, University of Coimbra, Coimbra, Portugal; † Centre for Innovative Biomedicine and Biotechnology, University of Coimbra, Portugal; ‡ Faculty of Pharmacy, University of Coimbra, Coimbra, Portugal; § Department of Neuroscience, Chronobiology and Sleep Institute, Perelman School of Medicine, University of Pennsylvania, Philadelphia, Pennsylvania, USA; ‖ Institute for Interdisciplinary Research, University of Coimbra, Coimbra, Portugal; ¶ Howard Hughes Medical Institute, University of Pennsylvania, Philadelphia, Pennsylvania, USA

**Keywords:** *Drosophila*, clock genes, glia, circadian rhythms, sleep, metabolism, feeding rhythm

## Abstract

Rhythmicity is a central feature of behavioral and physiological processes, including sleep, immune responses, and metabolism. Research on brain control of these processes has largely focused on neurons, with less known about the role of clock genes in glial cells. In this study, we addressed the function of glial clocks by targeting the expression of key clock genes in glia of *Drosophila melanogaster.* Loss of the *period (per*) gene in glia increases sleep following aseptic injury and loss of either *per* or *timeless* (*tim)* significantly reduces locomotor activity in light:dark cycles and in constant dark, but other than this, the major effect of clock gene loss in glia is on metabolic function. We demonstrate that disruption of either *tim* or *per* in glia affects glycogen stores and reduces metabolic rate. Disruption of either *tim* or *per* in glia also affects rhythms of feeding and overall food consumption. Notably, these effects of clock disruption are mediated by distinct glial subtypes, especially cortex glia. We propose that the major role of glial clocks is in the control of energy homeostasis and metabolic rhythms, which likely also accounts for effects on locomotor activity. These findings link metabolism and behavior via circadian regulation in glia.

Glial cells are important cells in the nervous system that contribute to neurotransmission, neuroprotection, immune responses, synaptogenesis, neuroinflammation, neuronal metabolism, and metabolic homeostasis (Pawate and Bhat, 2008; Magistretti, 2011; Yang and Zhou, 2019; De Backer and Grunwald Kadow, 2022). In *Drosophila melanogaster*, as in mammals, glial cells are categorized into distinct classes (Awasaki et al., 2008; Doherty et al., 2009; Stork et al., 2012; Axelrod et al., 2023), including astrocyte-like glia, which are analogous to mammalian astrocytes (Hartenstein, 2011), cortex glia that envelop neuronal somata and surface glia, comprising perineurial and subperineurial glia (Awasaki et al., 2008), that serve similar roles to the blood-brain barrier (BBB) in mammals (Stork et al., 2008). Each subtype contributes to the complex architecture and function of the nervous system (Hartenstein, 2011).

Most animal behaviors, such as sleep, feeding and locomotion, display a circadian rhythm, adjusted to a ~24-hour period by circadian clocks in the central nervous system (Frank, 2015; Panda, 2016; Williams and Naidoo, 2020). Molecular clocks are found in neurons and glia (Damulewicz et al., 2022; Ewer et al., 1992; Long and Giebultowicz, 2018) and in mammals clocks in both cell types contribute to the maintenance of rest: activity rhythms (Brancaccio et al., 2017). Indeed, autonomous clocks in mammalian astrocytes are even sufficient for rest: activity rhythms (Brancaccio et al., 2019), but this is not known to be the case in *Drosophila*. While *Drosophila* glia are implicated in the regulation of sleep amount and also in circadian rhythms of rest: activity behavior (Suh and Jackson, 2007; Ng et al., 2011; Jackson et al., 2015; Ng and Jackson, 2015; Artiushin et al., 2018; You et al., 2018; Zhang et al., 2018; Li et al., 2023a), clock genes within glia are not required for this behavior (Ng et al., 2011; Delventhal et al., 2019; Damulewicz et al., 2022). A molecular clock in the BBB glia drives rhythms of efflux transport, thereby conferring a rhythm of brain entry on to small lipophilic molecules (Zhang et al., 2021), but the function of other glial clocks is debatable. Rhythms of many physiological processes require non-neuronal clocks, such as those in peripheral tissues (Barclay et al., 2012; Finger and Kramer, 2021), raising the possibility that clocks in glia also contribute to different aspects of physiology.

Considering the availability of advanced genetic tools, the relatively small number of glial cells, and the well-characterized glial populations in *Drosophila*, this organism serves as an exceptional model for uncovering novel aspects of glial involvement in circadian physiology (Edwards and Meinertzhagen, 2010). In the present study, we examined the role of glial clocks, specifically focusing on *per and tim*, in the regulation of a range of different outputs. While we report effects of glial clock gene expression on locomotor activity and sleep under some conditions, these do not appear to reflect the primary output of glial clocks. Our results show that oscillators located in glia are important for modulating metabolic function, which includes glycogen stores, metabolic rate, endogenous feeding rhythms and food intake.

## Materials And Methods

### Fly Stocks and Rearing

Female *Drosophila melanogaster* were used for all experiments, while both female and male *Drosophila melanogaster* were used for circadian rhythm experiments. All flies were raised on corn-meal medium as described on the Bloomington Drosophila Stock Center (BDSC) website (soy flour and light corn syrup-based) at 25 °C under a under 12-hour light:12-hour dark cycle, except in experiments that indicate otherwise. Approximately 3- to 7-day-old flies were used for all experiments. The lines repo-Gal4/TM3, Alrm-Gal4, NP2222-Gal4/cyo, NP6293-Gal4, 9-137-Gal4/SM6a, Moody-Gal4, and *tim*-Gal4 were used as described previously (Artiushin et al., 2018; Li et al., 2023a). The lines *UAS-tim.gRNA* (BDSC #90768), *UAS-per.gRNA (*BDSC #90769), *UAS-acp98.gRNA* (BDSC #90770), *UAS-Clk.RNAi* (VDRC 42834) were obtained from the Bloomington Drosophila Stock Center (BDSC), the Vienna Drosophila Resource Center (VDRC) or were available from the lab stocks. CRISPR and RNAi lines are in an Iso31 background. In all experiments, flies with glial CRISPR targeting of the *acp98* gene served as controls.

### Behavior Monitoring System

Flies were entrained for 3-4 days to a light-dark (LD) cycle at 25 °C and a relative humidity of 65%. The protocols of the sleep monitoring system (DAM system) were previously described (Hendricks et al., 2000; Shaw et al., 2000). Locomotor activity was recorded with a TriKinetics *Drosophila* Activity Monitor (DAM; http://www.trikinetics.com/) in an I36-LL Percival Incubator (Percival Scientific, Iowa, USA).

Sleep/activity parameters (total sleep, mean sleep duration, number of sleep bouts, and activity) were analyzed for each 24-hour period and averaged across 2 or more days, and the first day was discarded.

For each locomotor assay, groups of 20 to 32 flies per condition were loaded into multibeam DAM5H or single beam DAM2 monitors for each of the 2 to 4 experimental replicates. DAM2 monitors were specifically used for sleep deprivation and survival assays due to practical limitation of the multibeam monitors, as well as for tracking sleep and locomotor rhythms across different glia subtypes. In the activity graphs, *counts* refer to experiments conducted using the DAM2 system, which contains only one infrared beam, whereas *movements* refer to experiments conducted using the DAM5H system, which is equipped with 15 infrared beams. No notable behavioral differences were observed when genotypes were tested using both types of DAM monitors.

### Circadian Rhythm

For circadian rhythm analysis, flies were entrained for 3-4 days to an LD cycle at 25 °C and a relative humidity of 65% and then released into constant darkness (DD) at 25 °C for at least 5 days. Circadian rhythmicity was measured from locomotor activity consolidated into 15-min bins using FileScan software (v. 311, TriKinetics, Inc, Waltham, MA), and then analyzed using ClockLab software (Actimetrics). Rhythmicity and period length were determined from chi-square periodograms at *p* < 0.01.

### Sleep Deprivation

Sleep deprivation was performed using up to four DAM2 monitors attached to a multi-tube vortexer (VWR) fitted with a mounting plate from TriKinetics, Inc. After 2 baseline days, flies were kept awake for 8 h (ZT16-0) with vibratory pulses applied for 1 second at random intervals with a mean duration of 20 s (O’Hara et al., 2024). Sleep gain and sleep loss were quantified as previously described (O’Hara et al., 2024). Sleep latency values (in minutes) were determined from the time of lights-on (for recovery sleep). Flies that were already asleep before these time points were excluded from this analysis. Data were processed using custom software, Insomniac 3 (Gardner et al., 2016; Miyazaki et al., 2022). Up to 16 flies per genotype were tested across 3 experimental replicates.

### Beam Position Analysis

Beam position activity of flies was analyzed using output data from FileScan (v. 311), selecting the Monitor Positions output data type. Unlike conventional sleep and locomotor activity analysis, where extra readings are summed, this analysis averaged extra readings and grouped them into 1 h time bins. FileScan was used to consolidate “Pn” data into these hourly bins and exported as monitor output files and analyzed manually in Microsoft Excel.

The flies’ positions within the monitoring system were tracked across 15 distinct infrared beams, numbered from 1 to 15, with the food source positioned at location 1. The median daily position and the percentage of flies at position 1 for each genotype were also subsequently calculated using Excel. This data was then transposed into a new sheet, with each row representing an individual fly’s activity. A chart was created to illustrate the median positional data across time for the different groups of flies. Troughs in the actograms indicate the times when flies were at the food end, while peaks represent movement toward the opposite end or intermediate positions. It was essential that the FileScan software averaged the “Pn” values into the designated time bins to capture the variability in activity between the 15 positions over time. The percentage of flies at position 1 was calculated by determining the proportion of flies at position 2 or lower (<2) for each hour.

### Sterile Injury

To prepare the injection solution, 1% food coloring (#1 Brilliant Blue FCF) was diluted in phosphate-buffered saline (PBS). Two to three days after loading the flies into activity monitors, they were removed and anesthetized with CO_2_. The flies were then subjected to aseptic injury by injecting the food coloring solution in PBS using glass capillary needles.

Sleep responses were quantified as described previously (Kuo et al., 2010). Briefly, sleep induction was calculated in individual flies as the difference in sleep (minutes/6 h) between the day after injury and the equivalent time interval the day before injury. These differences were adjusted by subtracting the average difference for corresponding timepoints obtained from a handled control group. Flies that died within 24 h posttreatment were excluded from the analysis.

### Feeding Assay

Briefly, female flies aged 4-10 days were entrained at 25 °C in LD for 3 days. The flies were switched from normal food to blue-dye food (100 mL of 5% sucrose + 2% Agar food containing 2.5 g of FD&C Blue #1 25 G CAS3844-45-9 [Spectrum]) for 4 h at different time points, as previously described (Edgecomb et al., 1994; Xu et al., 2008). After each feeding period, flies were immediately collected using dry ice. Fly heads were removed and groups of 5 flies were homogenized, and the absorbance was measured at 625 nm by SpectraMax iD5. The feeding levels were also normalized to the absorbance value/no. of flies. Each condition has 4 to 6 biological replicates. The food intake was calculated by summing of the intake of all 6 points of individual flies (He et al., 2023). The measurements of feeding rhythm and feeding consumption are separately measured.

### Triglyceride and Glycogen Measurement

For each independent experiment, 20-30 female flies were divided into two to three groups, each consisting of 10 flies, as described. Flies were collected at two timepoints: ZT4 (daytime) and ZT18 (nighttime). Prior to collection, they were starved for 1 h. Approximately 10 fly bodies and 10 fly heads per biological replicate were collected in a 2-mL collection tube using dry ice, and the samples were lysed in 100 µL NP40 diluted in H_2_0 miliQ water (Cayman, 10010303) for 2 min at 25 Hz using the Tissue Lyser II (Qiagen). The homogenates were spun down, transferred to a new 1.5-mL collection tube and then centrifuged at 13,000 rpm for 10 min at 4 °C, and the supernatant was collected for subsequent measurement. Protein measurement was performed using a bicinchoninic acid (BCA) protein assay kit (Abcam, AB207003) according to the manufacturer’s instructions. Protein concentrations were used to normalize triglyceride and glycogen levels in different samples. The triglyceride (TAG) assay was performed using the TAG colorimetric assay kit (Cayman, 10010303) according to the manufacturer’s instructions.

For the glycogen assay, glycogen was first reduced to glucose using amyloglucosidase (Sigma, A1602-25MG) following the manufacturer’s instructions from the Glucose (GO) Assay Kit (Sigma, GAGO20), and as previously described (Li and Tennessen, 2017; Tennessen et al., 2014). Briefly, serial dilutions of glucose and glycogen standard stock solutions (both at 1 mg/ml) were prepared in phosphate buffer solution (PBS). A volume of 30 µl of glucose or glycogen standards, diluted in PBS, was loaded in duplicate rows onto a flat, clear bottom 96-well plate. One row was used to measure glycogen + glucose (treated with amyloglucosidase) and the adjacent row was used to measure glucose alone (no amyloglucosidase). Samples were diluted in PBS and 30 µl of each sample were loaded. Then, 100 µl of assay reagent were added, consisting of Glucose Oxidase (GO) reagent + enzyme + amyloglucosidase for glycogen readings and of GO + enzyme for glucose alone. The plate was incubated for 30 min at 37 °C. After incubation, 100 µl of 6 M H_2_SO_4_ was added as a stop solution. The plate was briefly shaken, and the absorbance at 540 nm was recorded using a SpectraMax iD5 plate reader. Results were normalized to protein content, and free glucose values were subtracted from the total glycogen + glucose readings to determine glycogen levels. Each experiment was performed at least 3 times.

### Analysis of Metabolic Rate

The metabolic rate was determined according to the method described by Brown et al. (2022). All experiments were conducted with groups of 25 to 30 female flies placed into behavioral chambers containing a food medium of 2% agar and 5% sucrose. CO_2_ output was measured over a 24-hour period. Flies were loaded approximately at ZT0, and for data analysis of total CO_2_ and O_2_, the first and last hours of data collection were discarded. Therefore, the metabolic rate was assessed by quantifying the amount of CO2 produced in 15-min intervals, considering only the period from ZT6 to ZT18. The initial hours of data collection were discarded to allow the flies to acclimate to the new setup, and the final hours were excluded to ensure an equal number of hours were analyzed for both the daytime and nighttime periods. The amount of produced CO_2_ (µL/h/fly) was normalized to the number of flies. Data were acquired with MAVEn software and processed with SSI ExpeData data acquisition and analyzes software (SSI v. 1.9 27) and R studio (v. 2023.12.1+402).

### Analysis of Starvation Sensitivity

Flies were loaded in glass DAM locomotor tubes containing 2% agar that maintains internal humidity and serves as a water source. Flies are considered dead from the time when all activity counts permanently reach zero in the assay, and survival time per fly calculated using the Insomniac3 software (Gardner et al., 2016; Miyazaki et al., 2022). Data are presented as the percent survival of cohorts, calculated based on the average survival time.

### Quantification and Statistical Analysis

Analyses were performed using Microsoft Excel or GraphPad Prism 9, and graphs were plotted using Prism 9.9.0. For comparison of survival curves, pairwise log rank (Mantel-Cox) analysis was used. For comparison among different genotypes or treatment groups, pairwise analyses were conducted by unpaired *t* test for two groups of data. Non-parametric tests were used for data that did not fit a normal distribution as determined by the Shapiro-Wilk test. Two-way ANOVA (or mixed model) was used to evaluate time vs genotype variable effects of groups of data. Statistical details of experiments can be found in the figure legends. *p < 0.05, **p < 0.01, ***p < 0.001, ****p < 0.0001, n.s., not significant. Data are represented as the mean ± SEM unless otherwise noted.

## Results

### Loss of Glial Clocks Reduces Locomotor Activity

**Figure 1.**
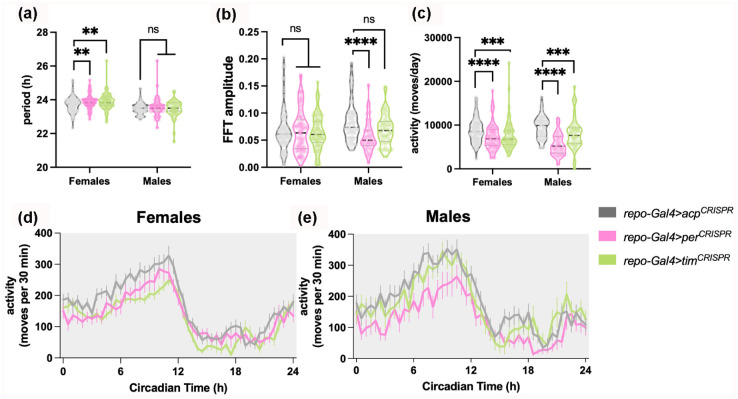
Loss of glial *per* or *tim* decreases activity levels and reduces locomotor rhythm strength in constant dark conditions. Violin plots showing the median and quartiles of (a) period length (h), (b) rhythm strength or amplitude as measured by FFT, and (c) activity moves per day (24 h) of female and male flies lacking *per* or *tim* in glia (Repo + cells) in comparison with controls *repo-Gal4* *> acp*^*CRISPR*^ group. (d, e) Mean ± SEM locomotor activity moves per 30 min. Significant alterations were evaluated using Mann-witney *t* test to compare medians with control *repo-Gal4* *> acp*^*CRISPR*^ group (**p < 0.01, ***p < 0.001; ****p < 0.0001; n.s., not significant). *N* = 90-96 per group for females and *N* = 40-61 per group for males across two to three experiments.

The role of glial cells in modulating circadian rhythms has been well-established in both mammals and flies (Jackson et al., 2015; Chi-Castañeda and Ortega, 2017). However, when clock gene expression is disrupted in glial cells, either by ablating *per* or *tim* expression, the effects on circadian rhythms and period length of flies are minimal (Ng et al., 2011; Delventhal et al., 2019). A previous study using flies showed that glial clock genes are important for the regulation of sleep amount and are essential for sustaining diurnal variations in the arborizations of circadian neurons (Damulewicz et al., 2022) and recent work suggested a role for glial clocks in rebound following sleep deprivation (Dopp et al., 2024). In this study, we first sought to clarify the effects of glial clock knockout on circadian rhythms and sleep. We used a pan-glial driver (*repo-Gal4*) to perform tissue-specific knockout of the core clock genes *per* and *tim* by employing Clustered Regularly Interspaced Short Palindromic Repeats (CRISPR)-Cas9 gene-editing tools, targeting previously validated guide RNAs (*repo-Gal4* *>* *per*^*CRISPR*^ and *tim*^*CRISPR*^) (Delventhal et al., 2019). The *accessory gland protein 98AB* (*acp*) was also targeted and used as the control (*repo-Gal4* *>* *acp*^*CRISPR*^) (Delventhal et al., 2019). Our results show that disrupting *per* or *tim* in glial cells of female flies slightly increased period length under constant darkness (DD) conditions but did not affect the amplitude of rhythm strength (FFT). Male flies showed a significant reduction in rhythm strength (FFT) with *per* knockout in glia, but no effect on period (Figure 1a and 1b). Notably, overall activity was significantly reduced in female and male flies lacking *per* or *tim* in glia relative to the *acp*-targeted control (*repo-Gal4* *> acp*^*CRISPR*^*)* (Figure 1c - 1e). In 12:12 light:dark (LD) cycles, disruption of *per* or *tim* in glia (Repo + cells) did not affect sleep or sleep architecture (Suppl. Fig. S1A-S1E). However, as in constant dark, daily locomotor activity was significantly reduced, as awake *per* and *tim*-deficient flies were both less active than control (*repo-Gal4* *> acp*^*CRISPR*^) (Suppl. Fig. S1B and S1F).

Prior studies have implicated glia (Blum et al., 2021), glial clock-controlled genes (You et al., 2021), or glial clocks (Dopp et al., 2024) in the regulation of sleep homeostasis. Although glial *per* and *tim* knockouts showed little to no effect on daily sleep, we sought to determine whether the glial *per* and *tim* knockouts alter the response to sleep loss. Flies were sleep-deprived for 8 h using mechanical stimulation, starting at ZT16 (ZT, zeitgeber time) and concluding at the beginning of the following day, at ZT0. Recovery sleep after mechanical sleep deprivation was similar between all groups, with no significant changes in sleep gain or sleep latency, indicating that glia clocks are not required for homeostatic sleep rebound (Suppl. Fig. S1G-S1L).

To validate the efficacy of the CRISPR constructs used in our study, we performed control experiments targeting *per*, *tim*, and *acp* in *tim* *+* clock neurons using the *tim-Gal4 driver*. Consistent with previous studies (Delventhal et al., 2019), disruption of *per* or *tim* in *tim*^+^ cells led to arrhythmicity in DD (Suppl. Fig. S2A). We observe that *tim-Gal4* *>* *per*^*CRISPR*^ led to almost complete loss of rhythmic behavior (9% and 11% rhythmic males and females, respectively), while *tim-Gal4* *>* *tim*^*CRISPR*^ led to complete loss of rhythmic behavior (no rhythmicity whatsoever) (Suppl. Fig. S2B).

In contrast, control flies (tim-Gal4 > *acp*^*CRISPR*^) maintained circadian locomotor activity (100% and 94% rhythmic males and females respectively) and show a ~24 h period (Suppl. Fig. S2B).

These data confirm that the *per* and *tim* CRISPR constructs are functional and capable of effectively disrupting circadian rhythmicity when targeted to canonical clock neurons. The milder circadian phenotypes observed in glial knockouts are therefore not due to ineffective gene targeting but likely reflect a limited role of glial clocks in rhythmic behavior. We conclude from these findings that glial clocks are important for maintaining normal locomotor activity levels with minimal effects on circadian rhythms and sleep regulation.

### Injury-Induced Sleep Duration Is Modulated by *per* Expression in Glia

**Figure 2.**
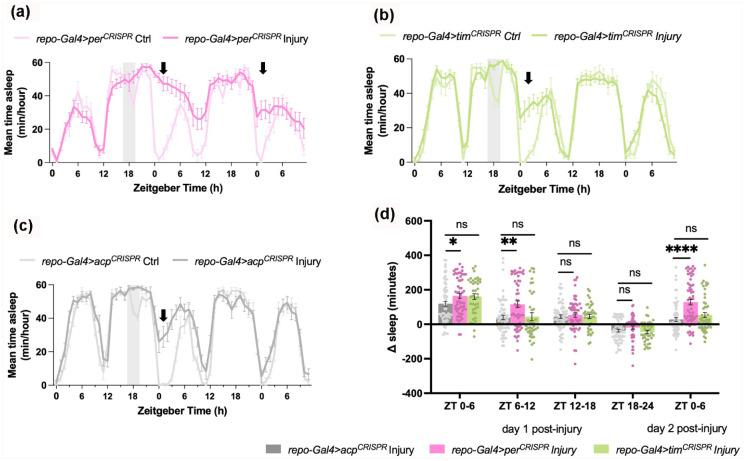
Disruption of glial *per* promotes prolonged increase in sleep following sterile injury. (a —c) Representative results from sterile injury (Injury) at ZT18 (shaded area) in *repo-Gal4* *> per*^*CRISPR*^, *repo-Gal4* *> tim*^*CRISPR*^, and control *repo-Gal4* *> acp*^*CRISPR*^, respectively. Mean ± SEM time asleep (minutes/hour) is plotted for 3 consecutive days in 1-h increments. Note increase in sleep the second morning after treatment (arrow). (d) Mean ± SEM net changes in sleep are reported from ZT0-6 the morning after treatment in 6-hour increments (ZT0-6, 6-12, 12-18, 18-24, and ZT0-6 on the second day postinjury). Values for mean net changes in sleep in (d) are normalized to those in handled control groups (Ctrl) (see “Methods”). Significant alterations were evaluated using unpaired parametric *t* test for comparisons with control *repo-Gal4* *> acp*^*CRISPR*^ injured group (*p < 0.05, **p < 0.01, ***p < 0.001; ****p < 0.0001; ns, not significant) *N* = 48-69 flies per group across three to four replicate experiments.

Sleep is a complex behavior influenced by various factors, including different stressors (Williams and Naidoo, 2020). For example, sleep is essential for recovery from acute stressors or illnesses, such as ultraviolet (UV) radiation, exposure to toxins, pathogenic infections, and mechanical damage, by promoting cellular adaptations that enhance recovery (Kuo et al., 2010; Suchecki et al., 2012; Hill et al., 2014; Lenz et al., 2015; DeBardeleben et al., 2017; Toda et al., 2019; Goetting et al., 2020; Singh and Nandave, 2023). Glial cells play a key role in neuroimmune responses, with growing evidence highlighting a link between sleep and glial-mediated immune functions (Ingiosi et al., 2013; Irwin, 2019; Williams, 2019). To clarify the functions of glial *per* and *tim* in stress-induced sleep by sterile injury, flies were monitored for two baseline days and sterile injury was performed on the third day at ZT18 in a 12: 12 LD cycle. This specific timepoint was chosen based on findings that infection and aseptic injury at nighttime (ZT18) significantly increase morning sleep (Kuo et al., 2010). Sleep was continuously monitored for at least two days following injury. In addition, a handled control group (Ctrl) was subjected to the same handling, including removal from the activity monitors and CO_2_ anesthetization for the same duration, without the injury. All groups showed increased sleep relative to the corresponding handled control group in the morning following injury, which typically corresponds to an arousal period in control or undisturbed flies as it is the daytime start period (Figure 2a-2c). Specifically, a robust and prolonged increase in sleep was observed from ZT0-6 in all flies. However, *repo-Gal4* *>* *per*^*CRISPR*^ flies also showed a significant increase in sleep during the ZT0-6 morning period on the second day postinjury. *tim knockout* flies (*repo-Gal4* *>* *tim*^*CRISPR*^) were not significantly different from controls or from *per* (Figure 2d).

To evaluate whether glial clocks influenced the timing of injury-induced sleep, we tested the response to injury administered in the daytime, at ZT6. Sleep in response to injury at ZT6 was previously reported to be weak or absent, in contrast to that at nighttime (Kuo et al., 2010). While all flies showed an increase in sleep immediately following treatment at ZT6, no effect on sleep was noted during the next morning period, with no significant differences between genotypes (Suppl. Fig. S3A-S3D).

In summary, these findings indicate a non-circadian role of glial *per* in limiting the duration of injury-induced sleep. Given the observation that loss of glial clocks reduced daily locomotor activity, but with limited effects on daily and stress-induced sleep, we next evaluated a function in energy storage and metabolism.

### *per* and *tim* in Glia Regulate Energy Storage

Metabolic regulation is evolutionarily conserved between *Drosophila* and humans, with glycogen and triacylglycerol (TAG) serving as universal energy sources (Yamada et al.,2018, 2019; Heier et al., 2021). The catabolism of these reserves is required for key physiological functions, including embryogenesis, survival, immunity, and flight (Wigglesworth, 1949; Arrese and Soulages, 2010; Ganeshan and Chawla, 2014; Welte, 2015; Bland, 2023). Animals, including insects, accumulate TAG and glycogen to fuel metabolism during periods of food scarcity and increased energy demand (Gáliková and Klepsatel, 2023).

The synthesis and breakdown of energy stores must be tightly regulated, as dysregulation can lead to pathology (Ellingwood and Cheng, 2018; Gáliková and Klepsatel, 2023). Circadian modulation can be an important part of this regulation, and indeed in *Drosophila* glycogen is known to change with time of day (Zimmerman et al., 2004; Seay and Thummel, 2011).

To investigate whether the effects of reduced locomotor activity prompted by the knockout of clock genes in glia impacted energy storages, we analyzed the effects of the loss of *per* or *tim* in glia on energy reserves by measuring the levels of TAG and glycogen in the flies’ heads and bodies during the day (ZT4-6) and night (ZT18-20) under normal LD conditions. Heads and bodies were analyzed separately due to known differences in energy storage and metabolic activity between these compartments (Zimmerman et al., 2004).

**Figure 3.**
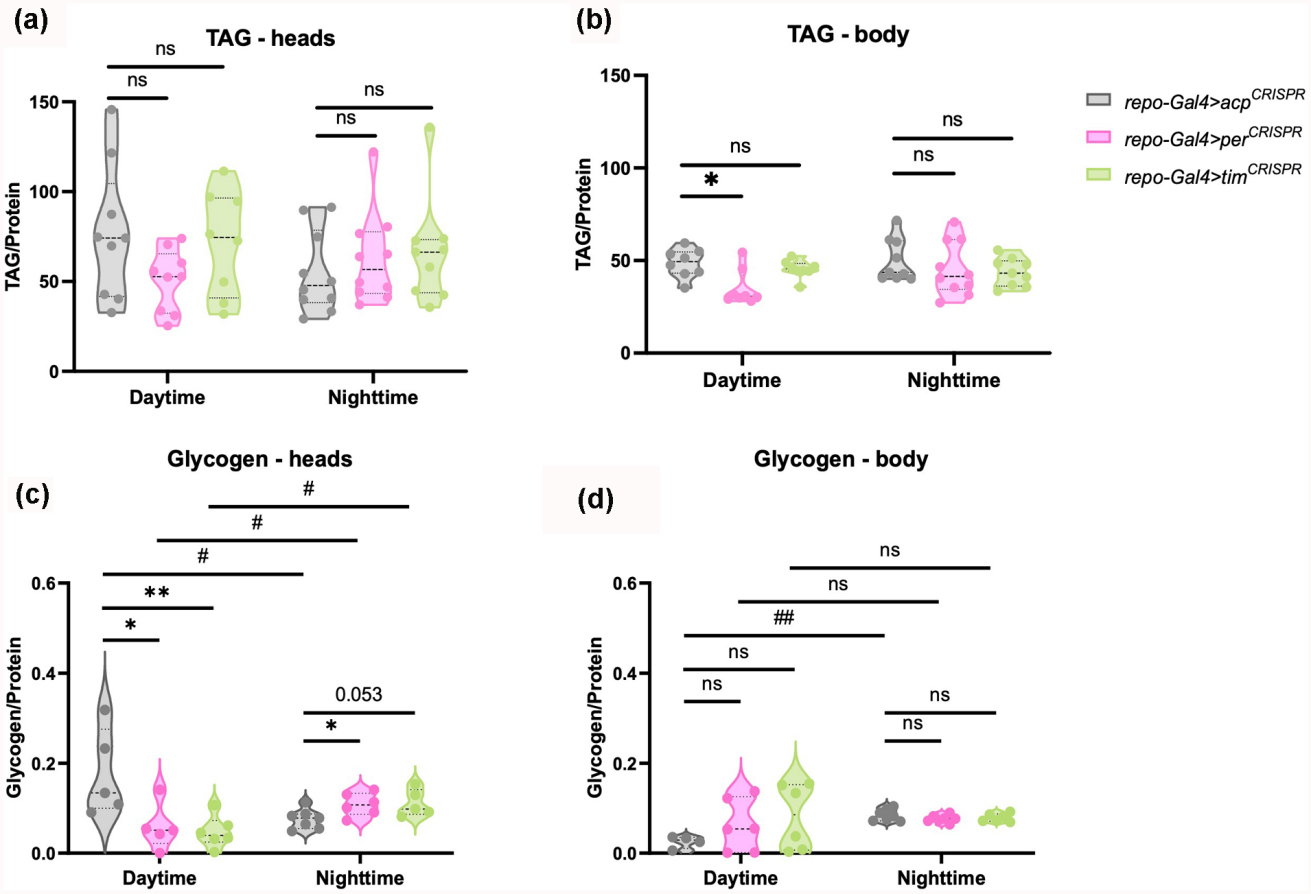
Loss of glial *per* or *tim* disrupts energy storage. Violin plots depicting the median and interquartile range of TAG (mg/dL) (a-b) and glycogen levels (mg/mL) (c-d) in the heads and bodies, respectively, of flies lacking *per* and *tim* in glia cells (*repo-Gal4* *> per*^*CRISPR*^, *repo-Gal4* *> tim*^*CRISPR*^, respectively) for daytime (ZT4-6) and nighttime (ZT18-20). *p < 0.05, **p < 0.01; ns, not significant. Mann-Whitney test was used for comparisons with the control *repo-Gal4* *> acp*^*CRISPR*^ group. ^#^*p* < 0.05, ^##^*p* < 0.01 ns, not significant for intra-group comparisons between day- and nighttime were also conducted using Mann-Whitney test. *N* = 8-10 biological replicates for TAG and 4-6 biological replicates for glycogen (10 fly heads or bodies per replicate).

The results show that TAG levels in the heads were similar between conditions, with no significant differences observed during either the daytime or nighttime (Figure 3a). However, TAG levels were significantly lower in the bodies of *repo-Gal4* *> per*^*CRISPR*^ flies compared with the controls during daytime, indicating reduced fat storage (Figure 3b).

Glycogen levels were lower during the day in heads of both *repo-Gal4* *> per*^*CRISPR*^ and *repo-Gal4* *> tim*^*CRISPR*^ than in control *repo-Gal4* *> acp*^*CRISPR*^ flies. At nighttime, the trend was reversed, with *repo-Gal4* *> per*^*CRISPR*^ expressing higher glycogen levels than the control *repo-Gal4* *> acp*^*CRISPR*^, and head glycogen levels in *repo-Gal4* *> tim*^*CRISPR*^ also trending higher (*p* = 0.053) (Figure 3c). As a result, glycogen levels in heads of both knockouts significantly increased from daytime to nighttime, while the control group’s glycogen levels significantly decreased during the night (Figure 3c). In the body, glycogen levels of *repo-Gal4* *> acp*^*CRISPR*^ increased from daytime to nighttime, but neither *repo-Gal4* *> per*^*CRISPR*^ nor *repo-Gal4* *> tim*^*CRISPR*^ glycogen levels showed a significant difference between the daytime and nighttime timepoints. Also, neither was significantly different from controls at either time. This was at least in part because of high variability in daytime glycogen amount in the knockouts (Figure 3d). Notably, protein concentrations used to normalize TAG and glycogen levels showed minimal variance across samples and so are unlikely to account for the observed differences (Suppl. Fig. S4a-S4b).

Overall, the disruption of glial clocks affects the regulation of energy reserves, particularly influencing glycogen levels in a compartment-specific and time-dependent manner, highlighting a relationship between glial clock function and metabolic homeostasis in *Drosophila*.

#### Clock Genes in Cortex Glia Regulate Metabolic Rate

**Figure 4.**
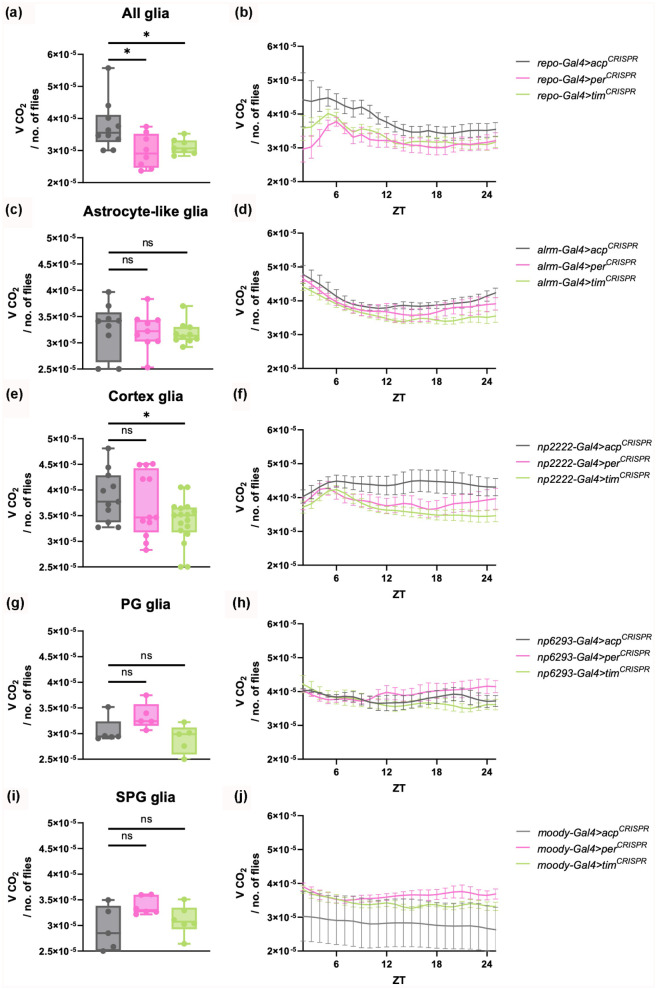
Disruption of *tim* in all glia, specifically in astrocyte-like glia and cortex glia, decreases overall metabolic rate. Box plots depicting the median and interquartile range of the CO_2_ volume produced between ZT6 and ZT18 by flies of the indicated genotypes are shown in the left panels (a, c, e, g and i). These data were extracted from the profiles of CO_2_ output across the entire day, shown in the right panels (b, d, f, h and j). Significant alterations were evaluated using Mann-Whitney test for comparisons with control *acp*^*CRISPR*^ group (*p < 0.05, **p < 0.01; ns, not significant). *N* = 5-10 biological replicates (25-30 flies/repeat) across three to four replicate experiments.

Both sleep and glial cells have been strongly correlated with metabolic regulation (Afridi et al., 2020; Haynes et al., 2024; Li et al., 2023a, 2023b; McMullen et al., 2023; Yurgel et al., 2015). Given the lower daytime glycogen storage observed in heads of the glial clock mutants, we speculated that the metabolic rate of these flies was affected. To investigate how energy expenditure is impacted by the disruption of *per* and *tim* in glia, we evaluated the CO_2_ output throughout the day (Figure 4a) as well as the average volume of CO_2_ produced during a segment of this time (ZT6-ZT18). The results showed that the average CO_2_ volume was significantly decreased in both *per-* and *tim*-deficient flies (Figure 4b).

Based on the data suggesting that oscillators located in glial cells play a role in metabolic rate regulation, we sought to identify specific types of glia responsible for this phenotype. We employed different drivers to manipulate gene expression in astrocyte-like glia (*Alrm-Gal4*), cortex glia (*NP2222-Gal4*), perineural (PG) glia (*NP6293-Gal4*), and subperineural (SPG) glia (*Moody-Gal4*). The average volume of CO_2_ produced was significantly reduced with *tim* disruption in cortex glia and also trended lower with *per* disruption in the same cells (Figure 4e, f). However, it was not affected by *per/tim knockout* in any other glial subtype (Figure 4). To determine whether this reflected clock function, as opposed to non-circadian effects of *tim/per*, we disrupted *Clock* expression in cortex glia and also observed a significant reduction in metabolic rate (Suppl. Figure S5A-S5B).

Furthermore, we monitored O_2_ output along with the average volume produced from ZT6 to ZT18 (Suppl. Fig. S6A-S6 J). As observed for CO_2_ volume, oxygen volumes trended lower with *per/tim* disruption in all glia, astrocyte-like glia or cortex glia, although these trends did not reach statistical significance (Suppl. Fig. S6A-S6 F).

As noted above, glial *per* and *tim* knockouts reduced daily locomotor activity levels in both constant dark (Figure 1d) and light:dark conditions (Suppl. Fig. S1G), suggesting that effects on activity may correlate with defects in metabolic rate. To determine whether this was the case, we recorded overall sleep and locomotor activity with knockout of *per/tim* in the different glial subtypes. There was a reduction of total activity with *tim*-knockout in all subtypes (cortex, PG and SPG glia) except for astrocyte-like glia, indicating that flies lacking *tim* in specific glia generally move less (Suppl. Figu. S7A-S7 H). Disruption of *per* in SPG glia (*moody-Gal4* *> per*^*CRISPR*^) also reduced total activity (Suppl. Fig. S7G-S7 H).

Knockout of clocks in the different glial subtypes resulted in subtle but significant changes in sleep. Knockout of *per* in astrocyte-like glia (*alrm-Gal4* *> per*^*CRISPR*^) led to a slight reduction in nighttime sleep relative to control *alrm-Gal4* *> acp*^*CRISPR*^ (Suppl. Fig. S8A-S8B), while knockout in cortex glia (*np2222-Gal4* *> per*^*CRISPR*^) caused a slight reduction in daytime sleep relative to controls (Suppl. Fig. S8C-S8D). Interestingly, *tim knockout* in cortex, PG or SPG glia increased nighttime sleep, likely due to reductions in overall locomotor activity (Suppl. Fig. S8C-S8H). No significant changes in daytime sleep were observed with knockout in PG glia (Suppl. Fig. S8E-S8F), but knockout of *per* or *tim* in SPG glia significantly increased daytime sleep (Suppl. Fig. S8G-S8H).

Taken together, these findings highlight a role for glial clocks in metabolic rate regulation. Reduced metabolic rate with knockout in cortex glia may be linked to decreased activity and increased nighttime sleep, although these features also result from *tim knockout* in PG/SPG glia without affecting metabolic rate.

#### *per* Expression in Cortex Glia Promotes Resistance to Starvation

Energy reserves and metabolic rate can determine the ability of an organism to survive/ resist starvation (Djawdan et al., 1998; Hoffmann and Harshman, 1999). To understand whether starvation resistance is related to glial clocks, we recorded survival rate under normal LD conditions during continuous food deprivation. Flies were kept on a 2% agar media to maintain hydration and humidity levels, as described in the “Methods” section. Given that the metabolic rate was affected by *tim* disruption in cortex glia (*NP2222-Gal4*) and showed similar trends for astrocyte-like glia (*Alrm-Gal4*), we focused on these cell types, along with knockout in all glia, to assay sensitivity to starvation. Data for each replicate experiment, including the number of flies per condition, the hazard ratio and statistical analysis, are shown in Supplemental Table S1.

**Figure 5.**
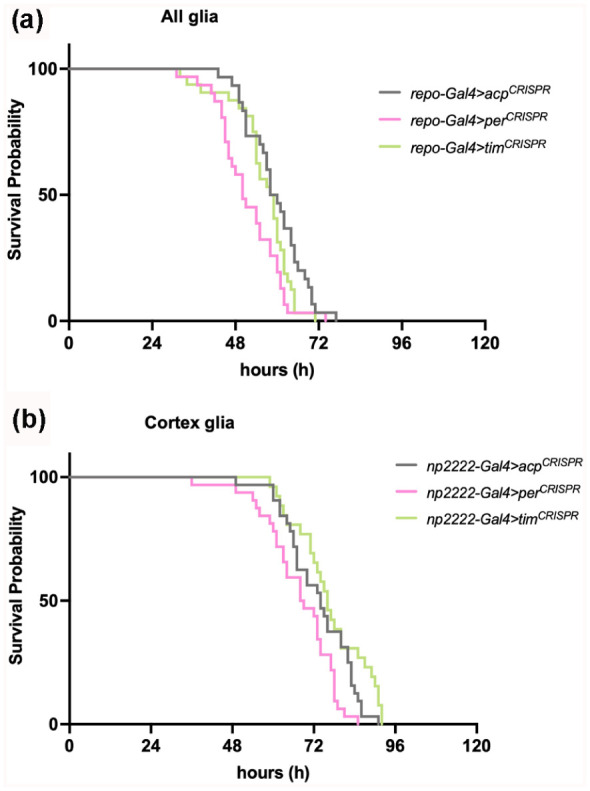
Disruption of *per* in cortex glia alters sensitivity to starvation. Representative Kaplan-Meier survival curves of starved flies with *per* and *tim knockout* in (a) all glia (*repo-Gal4*); and (b) cortex glia (*np2222*-*Gal4*). *N* = 26-32 flies per group.

The glial *per* knockout (*repo-gal4* > *per*^*CRISPR*^) significantly reduced survival to starvation in two out of three experiments and the third experiment also showed a trend toward reduced survival (Figure 5a and Suppl. Table S1). In addition, disruption of *per* in cortex glia, showed significantly reduced survival in three out of four experiments (Figure 5b and Suppl. Table S1). The results for astrocyte-like glia were variable, where both *per* and *tim* astrocytic knockouts (*alrm-gal4*) reduced survival from starvation in two out three experiments but went in the opposite direction in the third experiment (Supplemental Table S1). *tim*^*CRISPR*^ knockout in all glia and cortex glia also resulted in variable results across independent experiments. We conclude that despite glial clocks influencing locomotor activity and metabolic rate, they play a minor role in starvation resistance, with only cortex glial *per* modulating survival upon starvation.

#### Glial *per* and *tim* Regulate Feeding Rhythms

**Figure 6.**
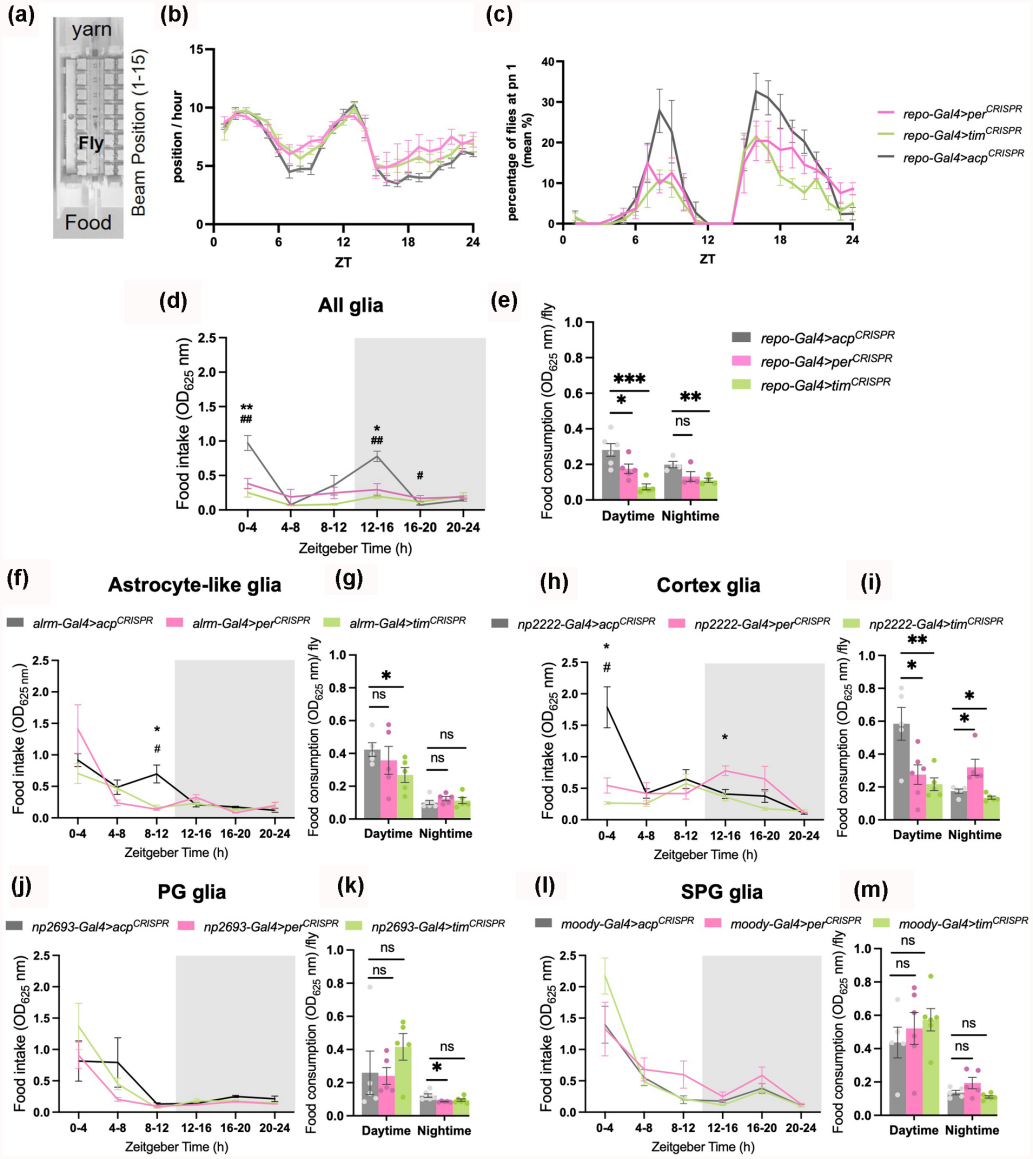
Glial clocks regulate feeding rhythms and food consumption. (a) Schematic of the monitor for beam position recording with position 1 (pn1) being closer to the food end and position 15 closer to the yarn. (b) Mean ± SEM position per hour of flies lacking *per* and *tim* in glia cells. (c) mean percentage of flies at the food end (pn1) per hour. *N* = 60-96 flies across 3 replicate experiments. (d-o) Mean ± SEM food intake per 4 hours and daily food consumption is shown in flies lacking *per* and *tim* in glia cells (*repo-Gal4*) (d-e), astrocytes-like glial cells (*alrm-Gal4*) (f-g), cortex glia (*np2222-Gal4*) (h-i), perineural glia (PG) (np2693-*Gal4*) (j-k), and subperineural glia (SPG) (*moody-Gal4*) (l-m), respectively. Two-way ANOVA was used to evaluate time vs genotype variable effects on food intake (*p < 0.05, **p < 0.01 for comparisons between *per*^*CRISPR*^ and control group *acp*^*CRISPR*^ and ^#^*p* < 0.05, ^##^*p* < 0.01 for comparisons between *tim*^*CRISPR*^ and control group *acp*^*CRISPR*^). Unpaired *t* test was used for comparisons of food consumption with control group *acp*^*CRISPR*^ (*p < 0.05, **p < 0.01, ***p < 0.001; n.s., not significant). *N* = 4-6 biological replicates (5 flies/repeat).

Previous studies have highlighted that both neuronal and peripheral clocks are necessary for regulating feeding (Fulgham et al., 2021; Xu et al., 2008). Our data implicating glial clocks in metabolic regulation prompted us to address their contribution to feeding rhythms. To clarify the functions of *per* and *tim* expression in glia in feeding rhythms, we first analyzed the position of flies relative to food during daily locomotor behavior in an LD cycle. During locomotor assays, flies are loaded into tubes containing 5% sucrose/agar as a food source and yarn at the other end. Multibeam monitors contain 15 beam positions, numbered from 1 to 15, allowing one to track the position of each fly every minute (Figure 6a). Given that the food is at position 1 (pn1), a lower number for the fly position reflects close proximity of the fly to the food end, while a higher number indicates the fly is at the yarn end (or the middle). As expected, flies show a rhythm of position preference. We find that flies with disrupted *per* or *tim* in glia tend to be further away from the food end both during the day and nighttime (Figure 6b). In addition, the percentage of flies at pn1 per hour is lower for both *per* or *tim* disrupted flies (Figure 6c). A similar pattern is observed in flies under constant DD, showing once again that mutant flies tend to be further away from the food end. However, the oscillations of position preference are less robust and pronounced than under regular LD conditions (Suppl. Fig. S9A and S9B).

Although flies with disrupted clocks in glia spend less time at the food end, the locomotor assay does not provide information on when they are feeding or how much food they are consuming. Thus, we measured the daily food intake at 4-h intervals in flies that were switched from normal food to blue-dye food to quantify the absorbance of consumed food in fly bodies (see “Methods” for details). The results showed that feeding rhythms are abolished with knockout of either *per* or *tim* in all glia (Repo + cells) (Figure 6d). In addition, we noticed that food consumption was changed in *per* and *tim*-deficient flies. Thus, we further quantified the food intake of individual flies during daytime and nighttime (see “Methods”). These data confirmed that food consumption was lower during daytime with glial disruption of either *per* or *tim* but during nighttime only *repo-Gal4* *> tim*^*CRISPR*^ was lower (Figure 6e).

To determine whether the role of *per* and *tim* in glia in feeding behaviors is specific to any of the glial subtypes, we next ablated these genes in astrocyte-like glia (*Alrm-Gal4*), cortex glia (*NP2222-Gal4*) and surface glia that consist of perineural (PG) and subperineural glia (SPG) (NP2693-*Gal4* and *Moody-Gal4*, respectively). *per* or *tim* ablation in cortex glia affected feeding rhythms (Figure 6h) but not in PG or SPG glia (Figure 6j and 6l). In astrocyte-like glia only ablation of *per* affected feeding rhythms. Knockout of *tim* in astrocyte-like glia reduced food intake during daytime (Figure 6g). Food consumption was also affected by knockout in cortex glia, wherein food intake was decreased during the daytime with *per* or *tim* disruption, but not during the nighttime, when *per* knockouts significantly increased their food intake (Figure 6i). Knockout of *per* or *tim* in PG glia reduced food intake during nighttime (Figure 6k). Food intake was not affected by clock disruption in SPG glia (Figure 6m).

Given that feeding rhythms and food intake were disrupted in *per* and *tim* cortex glia knockouts, we further investigated the impact of *Clk* downregulation in cortex glia on feeding-related behavior using position analysis. Flies with cortex glia-specific *Clk* knockdown (*NP2222-Gal4* *> clk*^*RNAi*^) exhibited altered positional preference rhythms, spending more time away from the food end during both the day and night (Suppl. Fig. S10A). In addition, the proportion of flies located at the food-adjacent position (pn1) per hour was lower in *Clk* knockdown flies compared to controls (*NP2222-Gal4* *>* *iso*) (Suppl. Fig. S10B.

Together, these findings reveal that the effects of glial clocks on metabolic rate are accompanied by changes in feeding rhythms and food intake, driven in particular by clock genes in astrocyte-like and cortex glia. Overall, the existence of circadian clocks in glia seems important for maintaining normal feeding behavior and energy homeostasis.

### Discussion

The findings presented here contribute to a growing body of evidence that glial cells play a crucial role in regulating a range of physiological processes. We found that the clock gene *per* acts in glia to affect injury-induced sleep and that glial clocks have important roles in energy metabolism, feeding, and locomotor behavior.

Glial cells play vital roles in neuronal development, activity, plasticity, and recovery from injury (Villegas et al., 2003; Parker and Auld, 2006; Li et al., 2020). An association between sleep and glial activation following neural injury has previously been suggested (Stanhope et al., 2020; van Alphen et al., 2022). We show here that flies lacking *per* in glial cells display a significant and prolonged increase in recovery sleep upon sterile injury, suggesting that *per* is normally required to curtail sleep at this time. While the reason for this is unclear, one possible explanation is that curtailing prolonged sleep during sickness provides an adaptive benefit, such as ensuring the flies remain awake to forage for food. Although prolonged sleep is generally associated with improved survival outcomes, these findings suggest that glial *per* might act to balance sleep needs with other survival behaviors during stress or injury. Additional studies of glial *per* knockout animals may reveal that they are compromised in some way by excessive sleep.

Our findings reinforce earlier studies indicating that circadian locomotor activity requires intact glial cells, yet does not depend on glial expression of clock genes (Ng et al., 2011; Delventhal et al., 2019). However, it is still possible that glial clocks contribute to activity rhythms. Although prior studies have disrupted glial clock oscillations or impaired glial signaling pathways, such as vesicular trafficking, calcium, or miRNAs (Damulewicz et al., 2022; Delventhal et al., 2019; Dopp et al., 2024; Ng et al., 2011; Ng and Jackson, 2015; You et al., 2018), specific manipulation of the period length of the glial circadian clock has not yet been explored and may offer novel insights into whether glia play a modulatory or instructive role in shaping behavioral rhythms.

We did not observe a significant effect of glial clock disruption on rebound after 8 h of mechanical sleep deprivation, which contrasts with a recent study by Dopp et al. (2024). This discrepancy could be explained, at least in part, by the different stimulation intensities and protocols used, given that we used a mild stimulation protocol over a shorter period, which may not have elicited sufficient sleep pressure to require glia-driven sleep regulation. In addition, while both studies employed CRISPR-mediated knockouts of *tim*, the other study knocked down other genes, including transcription factor gene *vrille (vri)* and *cryptochrome* (*cry*), while our experiments targeted *per* and *tim* expression. They also used a dominant negative version of the CYCLE transcriptional activator that could have ectopic effects. These factors, combined with the differences in experimental protocols, may account for the divergence in findings regarding sleep rebound after deprivation.

Prior research has shown that TAG levels in the fat body or whole body do not show circadian rhythms in wild-type flies or circadian clock mutants (DiAngelo et al., 2011; Seay and Thummel, 2011), but TAG levels are significantly reduced in circadian clock mutants compared to wild type flies (Seay and Thummel, 2011). In support of these findings, our results show that glial *per* mutant flies exhibit reduced daytime TAG levels in the body, without significant effects in the head. As previously suggested (Seay and Thummel, 2011), core clock genes may play a critical role in fat homeostasis, and perhaps the disruption of these clocks in glia affects TAG storage in the body. In addition, the absence of significant fluctuations between daytime and nighttime TAG levels further supports the notion that TAG levels in flies lack circadian rhythmicity, as previously reported (DiAngelo et al., 2011; Seay and Thummel, 2011).

Although previous studies have established that glycogen expression follows a daily pattern, there is considerable variation in the reported patterns (Zimmerman et al., 2004; Seay and Thummel, 2011). For instance, Seay and Thummel (2011) observed a 2-fold increase in whole body glycogen levels from ZT0 to ZT12 in wild-type flies, suggesting that a significant portion of consumed carbohydrates is stored as energy reserves (Seay and Thummel, 2011). In contrast, Zimmerman and colleagues (2004) reported significant diurnal variation in brain glycogen, with levels peaking during rest and declining during periods of activity, but saw no clear oscillation in glycogen levels in the whole head or body (Zimmerman et al., 2004). We observed differences in head (but not body) glycogen between day and night time points in control and glial *per/tim* CRISPR flies, with a phase consistent with that reported by Seay and Thummel (2011). In general glycogen regulation is complex, supported also by tissue-specific clock manipulations, which could contribute to differences across experimental systems. Clock disruption in the fat body reduces glycogen levels, while clock disruption in neuronal cells has the opposite effect (Xu et al., 2008). In addition, disruption of *gart*, a clock-controlled gene in glia, reduces glycogen levels in the fly throughout both day and night, without significant effects on TAG levels (He et al., 2023).

Flies lacking *per* or *tim* in glia show reduced glycogen levels in the heads relative to controls during the daytime and the opposite trend during nighttime, being significantly higher. This reversal suggests that glial circadian clocks play a role in shaping local glycogen rhythms, underscoring the importance of circadian regulation in glia for metabolic health.

A dynamic energetic balance is maintained through the regulation of energy intake (feeding), energy storage, and expenditure (metabolic rate), which are key determinants of longevity and resistance to adverse environments in *Drosophila* (Kühnlein, 2011; Katewa et al., 2016). Our data indicate that metabolic rate is reduced in flies lacking *per* and *tim* in glia. Specifically, cortex glia contribute to a reduced metabolic rate with *tim* and *Clk* disruption, while no effects were observed with astrocyte-like glia, PG and SPG glia. Although loss of *per* did not significantly affect metabolic rate, the trend was the same as with *tim*, suggesting that the regulation of metabolic rate is a glial clock-dependent phenomenon. Similarly, He et al. (2023) observed a reduced metabolic rate in flies lacking *gart* in glia (He et al., 2023), a clock gene, supporting our conclusion that glia influence metabolic activity through clock-regulated pathways.

Despite the decreased energy stores in flies lacking glial clocks, survival upon starvation was altered by knockout of *per* in all glia, and more specifically in cortex glia. This effect is consistent with previous findings showing reduced survival rate upon starvation in flies lacking the *gart* gene in glia; survival was restored upon *gart* re-expression (He et al., 2023). However, effects of the *tim knockout* in glia were variable, indicating a specific effect of *per*. Similar to the effects of *per* on injury-induced sleep, the effects on resistance to starvation are likely through non-circadian mechanisms, and independent of the metabolic phenotypes associated with glial *per/tim* knockout.

*per* or *tim* disruption in all glia reduces feeding and impairs feeding rhythms. The effect on feeding rhythms derives largely from cortex glia and astrocytes, and disruption in astrocytes, cortex glia or PG glia reduces food consumption. Earlier work showed that the clock in the fat body is important for regulating feeding rhythms (Xu et al., 2008). Fat body and glial clocks might be more important for controlling the strength of the feeding rhythm, while the central brain clock is the main driver of the period and timing of this rhythm (Fulgham et al., 2021).

Regarding sleep and activity, clock disruption in all glia reduces locomotor activity in conditions of 12:12 LD cycles and constant dark (DD). When looking at glia subtypes individually, knockout of *tim* in cortex glia, PG or SPG reduced activity and was associated with increased nighttime sleep; however, the sleep data showed variability across the different subtypes. These observations suggest that glial clocks modulate activity levels under constant conditions, while sleep regulation remains largely intact. However, our data contrast with previous evidence that shows that oscillators located in astrocyte-like glia, epithelial, subperineurial and chiasm giant glia are all involved in maintaining wake and sleep patterns, with specific effects on sleep parameters (Damulewicz et al., 2022). These differences could be partially explained by sex-specific effects as only males were used in the previous study. However, they may also be attributed to the use of different genetic tools, including thermogenetically controlled glia drivers, dominant negative transgenes, and RNAi for clock gene ablation (Damulewicz et al., 2022).

This study reveals the multifaceted role of glial clocks in regulating key physiological processes, including energy metabolism, feeding, and locomotor activity, emphasizing the importance of a well-organized network for maintaining homeostasis across different functions. The differential effects of clock gene disruptions in various glial subtypes underscore the complexity of glial contributions in maintaining energy balance and resilience under normal conditions as well as upon metabolic stress, indicating that different subsets may have unique, yet complementary, roles in sustaining circadian and metabolic functions. The observed effects on sleep and locomotor activity reinforce the notion that glial clocks contribute to modulating activity levels, although the mechanisms may differ from those implicated in sleep regulation.

### Study Limitations

Despite the significant insights provided by this study, several limitations should be acknowledged. First, although we demonstrated that *per* in glial cells regulates sleep after an immune challenge, and that glial clocks regulate energy homeostasis and feeding rhythms, the precise molecular pathways through which these genes exert their effects remain unclear. Although we identified a role for astrocyte-like and cortex glia in these processes, the functional heterogeneity of glial subtypes presents a challenge. Distinct glial populations appear to contribute differently to behavioral and metabolic regulation, and subtype-specific roles need to be fully resolved. More generally, we note that while our findings and those of others suggest a critical involvement of glial cells in maintaining rhythmic behaviors, differences in the experimental protocols and models used across studies may introduce variability. For instance, differences in sleep deprivation protocols, selection of glial cell drivers, and methods used to ablate clock genes, such as CRISPR-based tools versus dominant negative lines, can introduce variability in outcomes. In addition, a key factor contributing to variability may be the use of different glial Gal4 drivers, as these drivers differ in their spatial and temporal expression patterns, the strength of their expression and the backgrounds they derive from. These discrepancies underscore the need for standardized experimental approaches. At the same time, applying complementary tools to manipulate specific cell types and also to target gene expression, such as RNAi, dominant-negative constructs, and alternative core clock targets, can help reinforce the conclusions.

Finally, our study establishes a foundational framework for understanding the role of glial clocks in different rhythmic behaviors, but the complexity of glial signaling pathways suggests that further research is necessary to decode how glial clocks interact with neuronal circuits and systemic physiology. This would help to delineate the specific pathways by which glial clock genes regulate behavior and physiology under various environmental conditions.

## Supplemental Material

sj-docx-1-jbr-10.1177_07487304251386682 – Supplemental material for Regulation of Metabolic Rhythms by Glial Clocks Supplemental material, sj-docx-1-jbr-10.1177_07487304251386682 for Regulation of Metabolic Rhythms by Glial Clocks by Catarina Carvalhas-Almeida, Sara B. Noya, Tianqi Wu, Ana Rita Álvaro, Cláudia Cavadas, Julie A. Williams and Amita Sehgal in Journal of Biological Rhythms
