# Supplementary material for: Regulation of Metabolic Rhythms by Glial Clocks
Source: J Biol Rhythms. 2025 Dec 4;41(1):122–38. doi: 10.1177/07487304251386682 (PMC12694984; doi:10.1177/07487304251386682)
Supplement: sj-docx-1-jbr-10.1177_07487304251386682 – Supplemental material for Regulation of Metabolic Rhythms by Glial Clocks [file sj-docx-1-jbr-10.1177_07487304251386682.docx]

**Supplemental material for “Regulation of metabolic rhythms by glial clocks” in *Journal of Biological Rhythms*.**

**Supplementary Table**

**Supplementary Table 1 – Sensitivity to starvation effects on survival of flies lacking *per* or *tim* in different glia subtypes.** Replicate experiments flies lacking *per* or *tim* in all glia (*repo-Gal4*), astrocytes-like glia (*alrm-Gal4*) and cortex glia (*np2222-Gal4*) compared to controls *acp^CRISPR^*. n= number of flies per condition. A hazard ratio higher than one is indicative of higher mortality than the control group. In contrast, a ratio lower than one indicates higher mortality of the control group (*acp^CRISPR^*). Pairwise log rank (Mantel–Cox) analysis was used (*p < 0.05, **p < 0.01, ***p < 0.001; ****p < 0.0001; ns non significant).

| ***repo-Gal4*** |  | ***per^CRISPR^ vs acp^CRISPR^*** | ***tim^CRISPR^ vs acp^CRISPR^*** |
| --- | --- | --- | --- |
| **Experiment 1** | **n** | 37; 58 | 49;58 |
|  | **Hazard ratio (logrank)** | 1.521 * | 0.4449 **** |
| **Experiment 2** | **n** | 25; 32 | 26; 32 |
|  | **Hazard ratio** | 1.015 ns | 0.6921 ns |
| **Experiment 3** | **n** | 31; 30 | 32; 30 |
|  | **Hazard ratio** | 2.042 ** | 1.464 ns |
| ***alrm-Gal4*** |  | ***per^CRISPR^ vs acp^CRISPR^*** | ***tim^CRISPR^ vs acp^CRISPR^*** |
| **Experiment 1** | **n** | 31;32 | 32; 32 |
|  | **Hazard ratio** | 0.6916 ns | 0.6845 ns |
| **Experiment 2** | **n** | 32; 32 | 32; 32 |
|  | **Hazard ratio** | 2.042 ** | 1.464 ** |
| **Experiment 3** | **n** | 40; 61 | 59; 61 |
|  | **Hazard ratio** | 1.284 **** | 1.269 ** |
| ***np2222-Gal4*** |  | ***per^CRISPR^ vs acp^CRISPR^*** | ***tim^CRISPR^ vs acp^CRISPR^*** |
| **Experiment 1** | **n** | 32; 32 | 32; 32 |
|  | **Hazard ratio** | 1.655 * | 1.270 ns |
| **Experiment 2** | **n** | 32; 32 | 26; 32 |
|  | **Hazard ratio** | 1.757 * | 0.4784 ** |
| **Experiment 3** | **n** | 32; 32 | 32; 32 |
|  | **Hazard ratio** | 0.5790 * | 0.6205 * |
| **Experiment 4** | **n** | 32; 32 | 32; 32 |
|  | **Hazard ratio** | 1.757 * | 1.490 * |

**Supplementary Figures**

**Figure S1 - Loss of glial *per* or *tim* reduces locomotor activity without affecting sleep patterns or rebound after sleep deprivation.** (A) Representative experiment showing sleep per 30 minutes (mean ± SEM). (B) Activity per 30 minutes (mean ± SEM). Shaded area represents nighttime. (C) daytime and nighttime sleep, (D) sleep bout number, (E) sleep bout length, and (F) total activity moves per day of flies lacking *per* or *tim* in glia (Repo+ cells) in comparison with controls *repo-Gal4>acp^CRISPR^* group. Significant alterations were evaluated using unpaired t-test for comparisons with control *repo-Gal4>acp^CRISPR^* group (*p < 0.05, **p < 0.01, ***p < 0.001; ****p < 0.001, n.s., not significant). N = 59-87 per group across three to four experiments.

(G) Representative experiment showing sleep per 30 minutes (mean ± SEM). (H) Activity per 30 minutes (mean ± SEM). Shaded area represents the interval of sleep deprivation. Violin plots are shown for (I) sleep gain relative to corresponding baseline values during the 4 hours period (min) and (J) 12 h period (percentage, %), (K) percent sleep loss and (L) latency to first sleep bout following deprivation. The graphs show the median and quartiles. Significant alterations were evaluated using Mann-whitney t-test for comparisons with control *repo-Gal4>acp^CRISPR^* group (*p < 0.05, **p < 0.01, ***p < 0.001; ****p < 0.001, n.s., not significant). N = 46-66 per group across three to four experiments.

**Figure S2 – Loss of *per* or *tim* in clock neurons disrupts locomotor rhythm strength in constant dark conditions.** (A) Representative actograms of flies lacking *per* or *tim* in clock neurons (Tim+ cells) in comparison with controls *tim-Gal4>acp^CRISPR^* group (B) Table showing number of rhythmic flies per total number of flies (#R/n), % of rhythmic flies, period length and rhythm strength or amplitude as measured by FFT of female and male flies lacking *per* or *tim* in clock neurons (Tim+ cells) in comparison with controls *tim-Gal4>acp^CRISPR^* group.

**Figure S3 – Disruption of glial *per* or *tim* does not affect sleep following sterile injury during the daytime (ZT6)*.*** (A-C) Representative results from sterile injury (Injury) at ZT 6 (shaded area) in *repo-Gal4>per^CRISPR^*, *repo-Gal4>tim^CRISPR^* and *repo-Gal4>acp^CRISPR^*, respectively. Mean ± SEM time sleeping (in minutes) is plotted for 3 consecutive days in 1 h increments. (D) Mean ± SEM net changes in sleep are reported from ZT 6-12, 6 hours after treatment in six-hour increments (ZT 6-12, 12-18, 18-24 and ZT0-6 on the second day post-injury). Values for mean net changes in sleep in (D) are normalized to those in handled control groups (Ctrl) (see Methods). Significant alterations were evaluated using unpaired t-test for comparisons with control *repo-Gal4>acp^CRISPR^* injured group (t test; *p < 0.05, **p < 0.01, ***p < 0.001; ****p < 0.0001) N = 13-30 flies across two experiments.


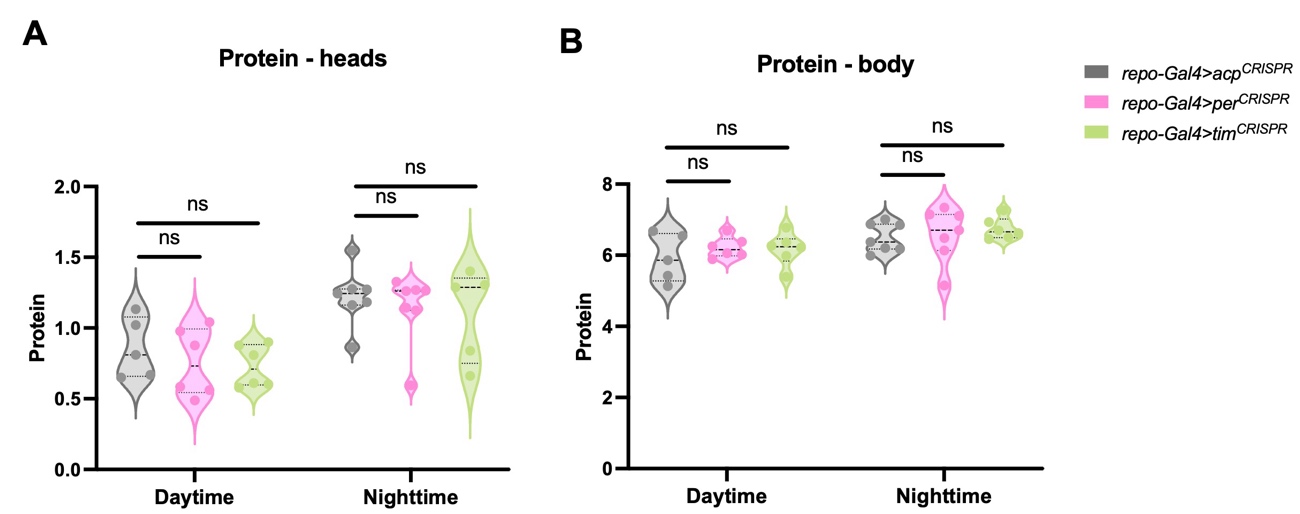


**Figure S4 – Protein amount of flies lacking glial *per* or *tim*.** Violin plots depicting the median and interquartile range of protein levels (mg/dL) (A-B) in the heads and bodies, respectively, of flies lacking *per* and *tim* in glia cells (*repo-Gal4>per^CRISPR^*, *repo-Gal4>tim^CRISPR^*, respectively) for daytime (ZT4-6) and nighttime (ZT18-20). ns, not significant. Mann-whitney test was used for comparisons with the control *repo-Gal4>acp^CRISPR^* group and for intra-group comparisons between day- and nighttime were also conducted using Mann-whitney test. N = 5-6 biological replicates (10 fly heads or bodies per replicate).

**Figure S5 – Disruption of *clk* in cortex glia reduces overall metabolic rate.** Profile of CO_2_ output of flies lacking *clk* in cortex glia is shown in the left panel (A). Box plot depicting the median and interquartile range of the volume of CO_2_ produced by flies lacking *clk* in cortex glia subtype is shown in the left panels (B). Significant alterations were evaluated using Mann-whitney test for comparisons with control *np2222-Gal4>iso* group (*p < 0.05, **p < 0.01; ns, not significant). N = 6 biological replicates (30 flies/repeat) across three replicate experiments.

**Figure S6 – Disruption of glial *per* or *tim* does not affect the volume of O_2_ consumed.** Profile of O_2_ output of flies lacking *per* and *tim* for indicated glia subtypes are shown in the left panels (A,C,E,G and I). Box plots showing volume of O_2_ consumed by flies lacking *per* and *tim* for indicated glia subtypes are shown in the right panels (B,D,F,H and J). Significant alterations were evaluated using Mann-whitney test for comparisons with control *acp^CRISPR^* group (*p < 0.05, **p < 0.01; ns, not significant). N = 5-6 biological replicates (25-30 flies/repeat) across 3-4 replicate experiments.

**Figure S7 – Loss of *tim* in cortex glia, perineural (PG) and subperineural (SPG) glia but not in astrocyte-like glia reduces activity levels.** Mean ± SEM activity counts per 30 minutes are shown in the left panels (A,C,E, and G), and quantified across the 24 hour period in the right panels (B,D,F, and H) for indicated glial subtypes. Significant alterations were evaluated using unpaired t-test for comparisons with control *acp^CRISPR^* group (*p < 0.05, **p < 0.01, ***p < 0.001; ****p < 0.0001; n.s., not significant). N = 20-70 per group across 2-3 replicate experiments.

**Figure S8 – Sleep amount across different glia subtypes lacking *per* or *tim*.** Mean ± SEM sleep per 30 minutes is shown in the left panels (A, C, E, and G), and quantified across the daytime and nighttime period in the right panels (B, D, F, and H) for indicated glial subtypes. Significant alterations were evaluated using unpaired t-test for comparisons with control *acp^CRISPR^* group (*p < 0.05, **p < 0.01, ***p < 0.001; ****p < 0.0001; n.s., not significant). N = 20-70 per group across 2-3 replicate experiments.

**Figure S9 – Disruption of *per* or *tim* in all glia affects feeding rhythms under constant darkness.** (A) Mean ± SEM position per hour of flies lacking *per* and *tim* in glia cells (B) mean percentage of flies at the food end (position 1, pn1) per hour, under constant darkness (DD). N = 60-80 per group across 3-4 replicate experiments.

** Figure S10 – Disruption of *Clk* in cortex glia affects feeding rhythms.** (A) Mean ± SEM position per hour of flies with *Clk* dysregulation in cortex glia cells (B) mean percentage of flies at the food end (position 1, pn1) per hour, under constant darkness (DD). N = 32 per group across 2 replicate experiments.
